# Synthesis of Cerium Oxide Nanoparticles in a Bacterial Nanocellulose Matrix and the Study of Their Oxidizing and Reducing Properties

**DOI:** 10.3390/molecules28062604

**Published:** 2023-03-13

**Authors:** Nina Melnikova, Darina Malygina, Vitaly Korokin, Hayder Al-Azzawi, Daria Zdorova, Evgeniy Mokshin, Elena Liyaskina, Irina Kurgaeva, Victor Revin

**Affiliations:** 1 Faculty of Chemistry, Lobachevsky University, 23/5 Gagarin Av., Nizhny Novgorod 603950, Russia; 2 Research Laboratory “New Polymeric Materials”, Nizhny Novgorod State Technical University n.a. R.E. Alekseev, 24 Minin St., Nizhny Novgorod 603950, Russia; 3 Department of Pharmaceutical Chemistry, Privolzhsky Research Medical University, 10/1 Minin Sq., Nizhny Novgorod 603950, Russia; 4 Department of Biotechnology, Bioengineering and Biochemistry, National Research Ogarev Mordovia State University, 68 Bolshevistskaya str., Saransk 430005, Russia

**Keywords:** bacterial cellulose aerogel, bacterial cellulose hydrogel films, nanobiocellulose ceria nanocomposite, cytochrome c, epinephrine, antioxidant

## Abstract

A soft synthesis of nanoceria with non-stoichiometric composition (33% Ce^3+^/67% Ce^4+^) named CeO_2_ NPs in bacterial cellulose (BC) matrix in the form of aerogel and hydrogel with controlled CeO_2_ NPs content was proposed. The advantage of CeO_2_ NPs synthesis in BC is the use of systemic antacid API–trisamine as a precursor, which did not destruct cellulose at room temperature and enabled a reduction in the duration of synthesis and the number of washes. Moreover, this method resulted in the subsequent uniform distribution of CeO_2_ NPs in the BC matrix due to cerium (III) nitrate sorption in the BC matrix. CeO_2_ NPs (0.1–50.0%) in the BC matrix had a fluorite structure with a size of 3–5 nm; the specific surface area of the composites was 233.728 m^2^/g. CeO_2_ NPs in the BC-CeO_2_ NPs composite demonstrated SOD-like activity in the processes of oxidation and reduction of cytochrome *c* (cyt *c*^3+^/cyt *c*^2+^), as well as epinephrine to inhibit its auto-oxidation in aqueous solutions by 33–63% relative to the control. In vitro experiments on rat blood showed a decrease in the MDA level and an increase in the activity of antioxidant defense enzymes–SOD by 24% and G6PDH by 2.0–2.5 times. Therefore, BC-CeO_2_ NPs can be proposed for wound healing as antioxidant material.

## 1. Introduction

Bacterial nanocellulose (BC) is a convenient polymer matrix for designing new drugs. In addition to a large specific surface area, controlled porosity, and moisture absorption, BC is biocompatible with respect to various types of mammalian cells and tissues, which makes it a promising material for tissue engineering [1,2], as well as for use in new composites for medical applications including those containing antibacterial components [3,4,5,6,7,8,9].

Reviews [10,11,12] show that BC composites with nanoparticles of metals such as silver, gold, platinum, palladium, cobalt, nickel, and copper, as well as metal oxides (zinc, titanium, iron, manganese, and others), have better antibacterial and physicochemical properties, including magnetic, photocatalytic, and sorption properties, compared to the original BC and can be used as drug delivery vectors, for example, cytostatics [13,14].

Recently, the inclusion of cerium oxide nanoparticles (CeO_2_ NPs) in nanocomposite materials based on BC or its composites with polysaccharides for medical use has been discussed in the literature [15,16,17,18,19,20]. In these works, special attention was paid to the aspects of tissue engineering, e.g., BC-based stem cell proliferation scaffolds, as well as antioxidant and enzymatic mimetic activities of CeO_2_ NPs in composites.

Progress in nanocomposite BC materials depends on both the nature and properties of BC, as well as on the unique physicochemical and pharmacological properties of CeO_2_ NPs, which exhibit powerful antioxidant and antibacterial effects [21,22,23]. In nanoparticles, cerium can exist either in a reduced (Ce^+3^) or completely oxidized (Ce^+4^) state, since it has partially filled 4f and 5d subshells [24].

The cerium atom can easily change its electronic configuration depending on the reaction conditions. The relative ratio of Ce^3+^ to Ce^4+^ cerium ions in particles depends on the particle size and pH [24]. The core of CeO_2_ NPs was shown to be enriched with Ce^+4^, and the surface is enriched with Ce^+3^, which is associated with oxygen vacancies or defects that ensure the ease of oxidation and reduction of cerium ions. With a decrease in the size of nanoparticles, the oxygen non-stoichiometry increases, and nanoceria acquires the properties of a wide-gap semiconductor. In this case, there is no strict relationship between nanodimensions and band gap. The instability of oxygen non-stoichiometry is due to rather easy Ce^3+^→Ce^4+^ and Ce^4+^→Ce^3+^ transitions. The non-stoichiometric nature of CeO_2_ NPs makes it possible for them to act as reduction or oxidation catalysts, depending on the medium [21,22,24]. Therefore, in this paper, nanoceria particles with a non-stoichiometric composition are referred to as CeO_2_ NPs, as is customary in the literature on nanoceria [18,19,20,21].

CeO_2_ NPs containing a high concentration of Ce^3+^ are of the greatest interest for medical use, since they provide a stable antioxidant effect required for the treatment of a number of diseases associated with ROS, including heart disease, Alzheimer’s disease, and cancer [25]. Unfortunately, despite the complex of useful pharmacological properties (antimicrobial, antioxidant, and others), the synthesis of CeO_2_ NPs in the BC matrix has not been extensively presented in the literature. Composites with BC and CeO_2_ NPs are usually obtained by mixing stabilized CeO_2_ NPs with polysaccharides, which frequently fails to obtain uniformly controlled distribution in the polymer matrix.

In the present work, we propose the synthesis of CeO_2_ NPs in a BC matrix to obtain BC-CeO_2_ NPs nanocomposites in the form of hydrogel films and aerogel powder with high concentration and uniform distribution of small CeO_2_ NPs in the BC matrix with antioxidant properties. The ability of CeO_2_ NPs and Ce^3+^ in the BC matrix to participate in redox reactions was studied using the example of interaction with cytochrome *c*, epinephrine, and ascorbic acid.

## 2. Results

### 2.1. Synthesis and Physicochemical Properties of Cerium Oxide Nanoparticles in a BC Matrix in the Form of a Hydrogel Film and Aerogel Powder

The physicochemical properties of CeO_2_ NPs synthesized in a bacterial cellulose (BC) matrix were compared with those of pure nanosized CeO_2_ NPs obtained by a modified procedure [26] using cerium (III) nitrate and 27% ammonium hydroxide at 60 °C in ethylene glycol–water mixtures.

**Figure 1:**
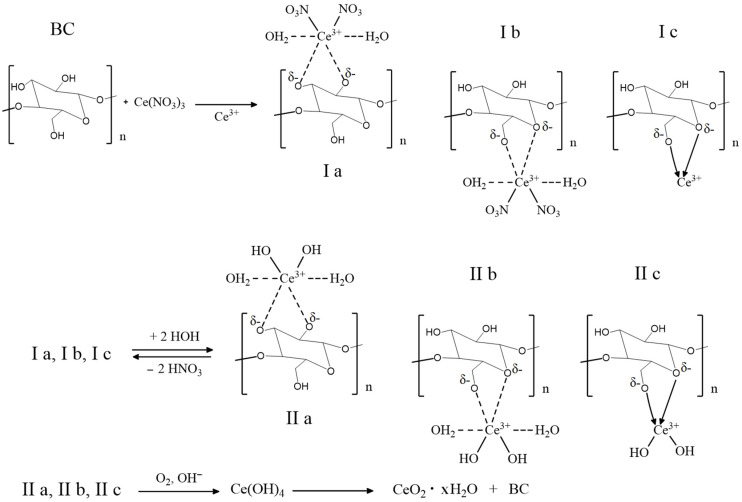
Schematic illustration of the synthesis of CeO_2_ NPs in a BC matrix.

In general, the schematic representation of the CeO_2_ NPs formation mechanism on the surface of BC probably corresponds to the general concept described by Karakoti A.S. (2007) [27]. Non-covalently-bound monomeric units of cellulose with cerium ions (structures Ia, Ib, and Ic) undergo hydrolysis to form products IIa, IIb, and IIc, followed by their oxidation to cerium (IV) hydroxide and hydrated CeO_2_ NPs in the BC matrix (Figure 1).

**Figure 2:**
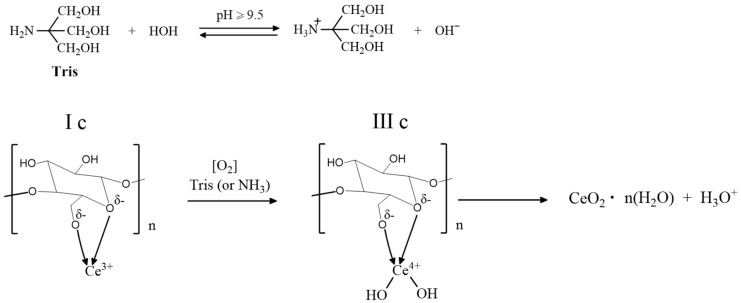
Schematic illustration of cerium (III) oxidation in a chelate complex with BC.

An alternative method for the precipitation of CeO_2_ NPs in the BC aerogel matrix is treatment of the reaction mixture with an aqueous 1 M trisamine solution (pH = 9–10) at room temperature for 30–60 min, which led to a decrease in the color of the product at all stages of preparation. Figure 2 shows the scheme of the proposed process. The same approach was used in the synthesis of BC-CeO_2_ NPs in a hydrogel film. Initially, a hydrogel film in a 0.9% NaCl solution was placed in a 1% solution of cerium (III) nitrate in ethylene glycol for 4–8 h to swell. After careful washings, the film was placed in an aqueous solution of trisamine for 10–30 min at room temperature until the film became stably yellow.

FTIR spectra, PXRD patterns, and EDX were used to identify CeO_2_ NPs in the BC matrix. The bands of Ce-O stretching vibrations (ν 450–570 cm^−1^) characteristic of the control sample of CeO_2_ NPs and the obtained samples of BC (aerogel)-CeO_2_ NPs and BC(hydrogel)-CeO_2_ NPs coincided (Supplementary Materials: Figure S1). In the FTIR spectra of BC-CeO_2_ NPs samples, the bands of stretching vibrations of CH_2_ and OH groups of ethylene glycol were observed (ν, cm^−1^: 1071.4; 3300–3400), as well as the bands of stretching vibrations of cellulose units (ν, cm^−1^: 596, 620, 642, 665, 744, 890, and 990 belong to stretching vibrations of 7 CH-OH and 1 CH_2_-OH and of alcohol C-O groups in the 1037–1200 region, as well as the intensive band in the region of 3355 cm^−1^), Figure S1.

**Figure 3:**
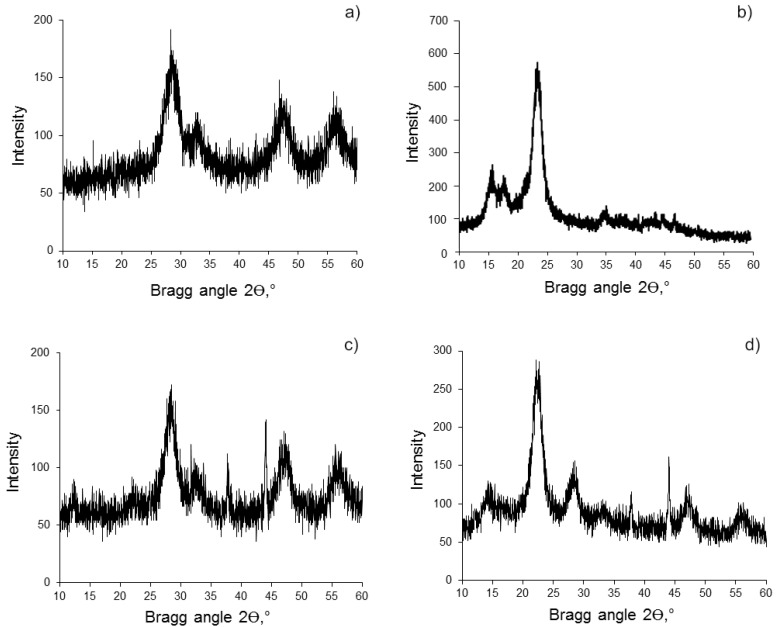
Powder XRD patterns of samples: (**a**) CeO_2_ NPs; (**b**) BC powder; (**c**) BC(aerogel)-CeO_2_ NPs; (**d**) BC(hydrogel)-CeO_2_ NPs. Signals at 2θ = 37° and 44° are due to the cuvette material.

The CeO_2_ phase in the resulting BC (hydrogel)-CeO_2_ NPs and BC(aerogel)-CeO_2_ NPs composites was established by the PXRD method (Figure 3, Table 1). The structure of CeO_2_ in the composites is similar to the fluorite-type cubic structure of the control CeO_2_ NPs, which is consistent with the powder XRD pattern of CeO_2_ in the ICDD-JCPDS database (JCPDS No. 34-0394, Figure S2).

Broad reflections in the PXRD patterns characterize the nanosize of cerium oxide both in the control sample and in the nanocomposites. The average crystallite size calculated on the basis of PXRD patterns data, in accordance with the Selyakov–Scherrer equation, is equal to 3.0–3.8 nm (Figure 3, Table 1).

The PXRD patterns also confirm the retention of the BC crystal structure and its nanosize in the BC(hydrogel)-CeO_2_ NPs and BC(aerogel)-CeO_2_ NPs composites. The signals in the region of the Bragg angles of 15–18° and 22°, as well as the ratio of their intensity, were preserved.

**Figure 4:**
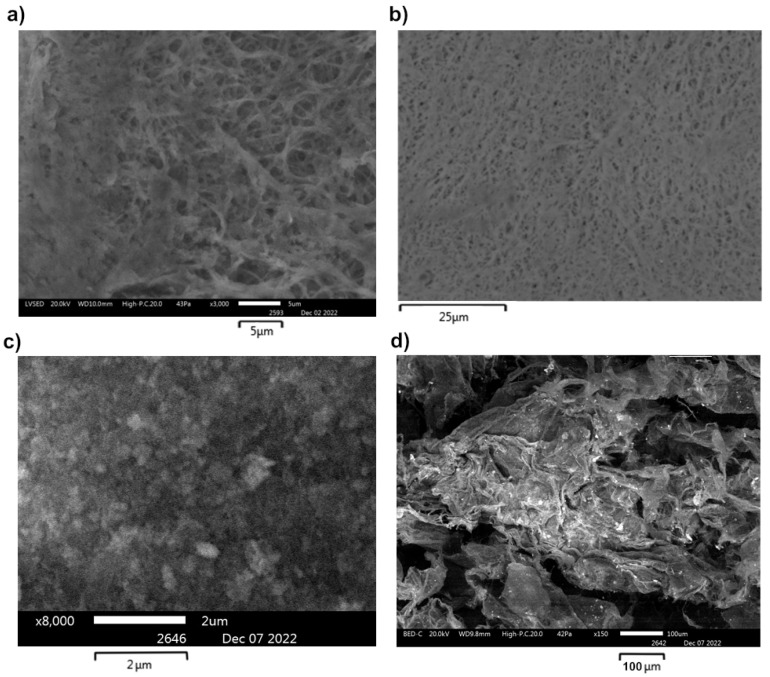
SEM images of BC(hydrogel)-CeO_2_ NPs (**a**,**b**) and BC(aerogel)-CeO_2_ NPs (**c**,**d**).

**Figure 5:**
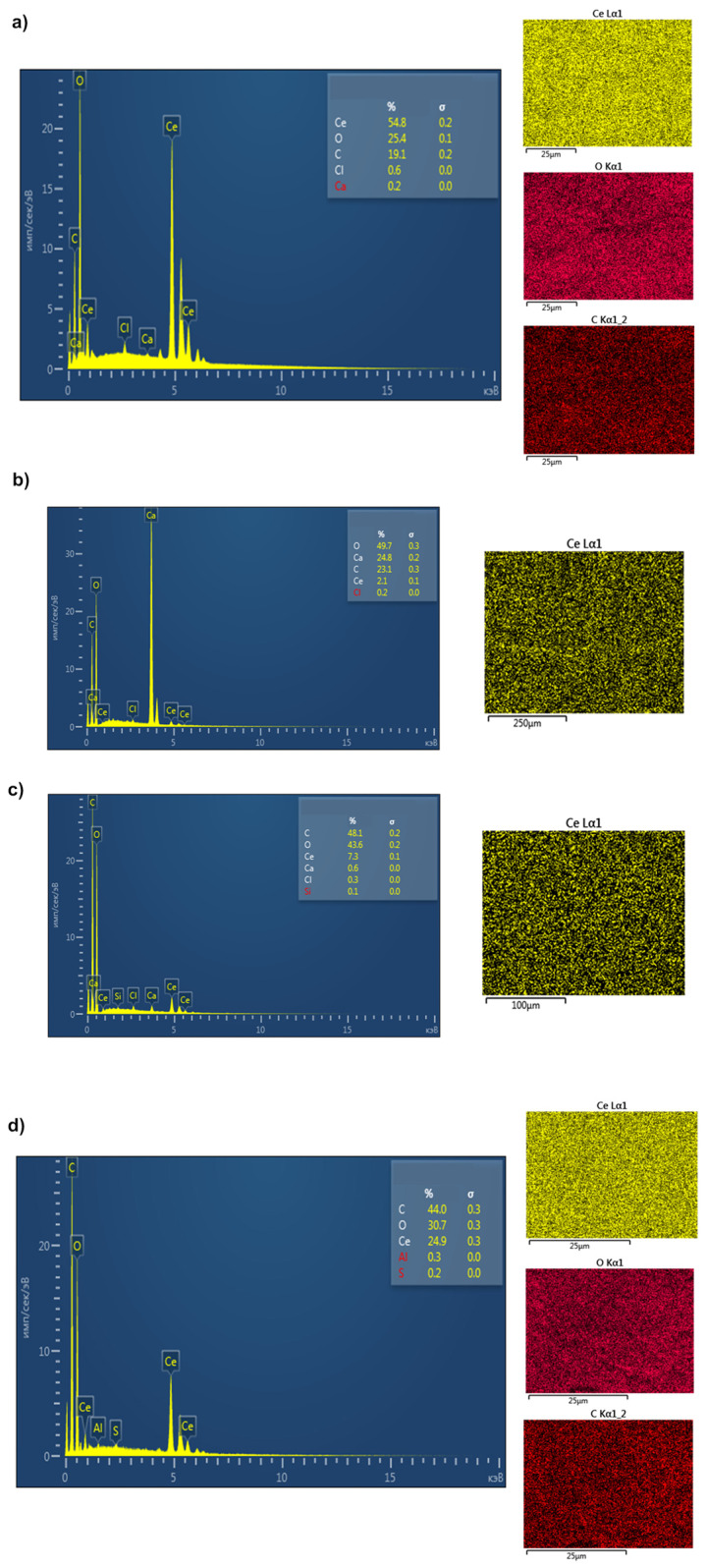
EDX data of BC(hydrogel)-CeO_2_ NPs (**a**–**c**) and BC(aerogel)-CeO_2_ NPs (**d**).

To study the film morphology and estimate the cerium concentration in the nanocomposites, we performed SEM and EDX studies of the samples and compared them with the control sample of CeO_2_ NPs (Figure 4, Figure 5 and Figure S3).

The concentration of CeO_2_ NPs varied from 2 to 55% in BC-CeO_2_ NPs nanocomposites, in accordance with EDX data. The specific surface area of powder was 233.728 ± 0.237 m^2^/g, the pore volume was 0.607 ± 0.015 cm^3^/g, and the pore diameter was 3.824 ± 0.028 nm, which indicates the high dispersity of the powder. These data are close to those of CeO_2_ NPs powder without BC (the specific surface area of the powder was 284.975 m^2^/g, the pore volume was 0.149 cm^3^/g, and the pore diameter was 1.120 nm) [28].

**Figure 6:**
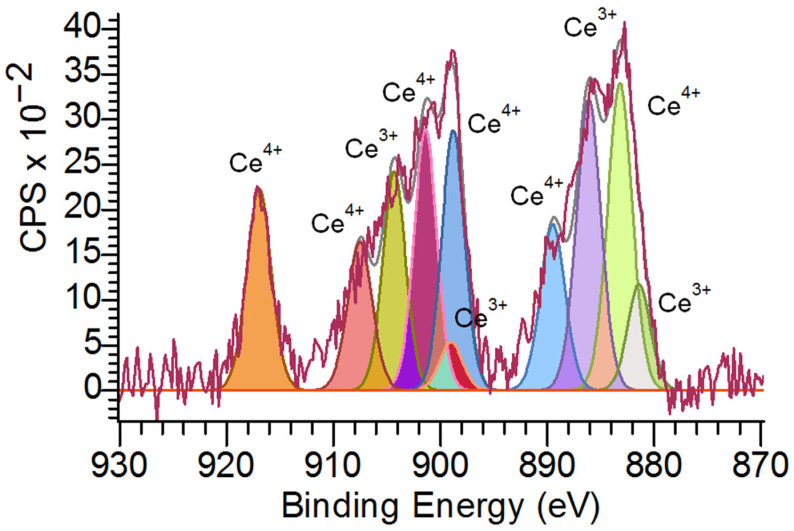
X-ray photoelectron Ce 3d spectrum of BC-CeO_2_ NPs.

Figure 6 shows that the Ce 3d XPS spectrum proved Ce^3+^ and Ce^4+^ valence states of cerium in BC-CeO_2_ NPs. The relative contents of Ce^3+^ and Ce^4+^ are equal to 33% and 67%, respectively, if CeO_2_ NPs are taken as 100%.

### 2.2. Reduction and Oxidation Processes under the Action of BC-CeO_2_ NPs

The participation of CeO_2_ NPs and BC-CeO_2_ NPs in the reduction process was also studied based on their reaction with a metalloprotein, cytochrome *c*, isolated from bovine heart serum in the oxidized form of cyt *c*^3+^. Cytochrome *c* (cyt *c*) plays an important role in the body due to electron transfer reaction [29,30]. It is expected that cyt *c* participates in electron transfer reactions with nanoceria particles with a stoichiometric composition close to that of CeO_2_ (Ce^4+^, 67%) in the core nanostructure and close to that of Ce_2_O_3_ (Ce^3+^, 33%) on the particle surface: Ce^3+^ + cyt *c*^3+^ ↔ Ce^4+^ + cyt *c*^2+^. On the surface of non-stoichiometric nanoceria under physiological conditions or in PBS, the cyt *c*^3+^ reaction easily proceeds only with Ce^3+^ ions.

We controlled the course of the cyt *c*^3+^ reduction reaction by UV-vis spectra, since the spectral properties of cyt *c* in different redox states are well-known and can be used as markers of its oxidated or reduced forms [31,32,33].

The reduced form of cyt *c*^2+^ was obtained by treating cyt *c*^3+^ with ascorbic acid in PBS. The choice of cyt *c* is due to both the importance of this metalloprotein in biochemical processes in the human body and the unambiguous difference between the oxidized and reduced forms in the UV-vis spectra. The reduced form of cyt *c*^2+^ has a Soret band (γ band) with λ_max_ = 414–415 nm and separated β and α bands with λ_max_ = 520 nm and λ_max_ = 550 nm, respectively. The oxidized form of cyt *c*^3+^ is characterized by a blue shift of the γ band from 415 nm to 408 nm and a diffuse band combining the α and β bands in the region of 520 nm [31,32,33].

Preliminary experiments showed that in the 0.3–0.7∙10^−5^ mol/L concentration range, the dependences A = f(C) for solutions of cyt *c*^3+^ and cyt *c*^2+^ correspond with the Bouguer–Lambert–Beer law (Figure S4), which enables assessment of the concentration of the oxidized and reduced forms both qualitatively and quantitatively using the absorption of the γ band and the band in the region of 520–550 nm (Figure S4, Table S1).

**Figure 7:**
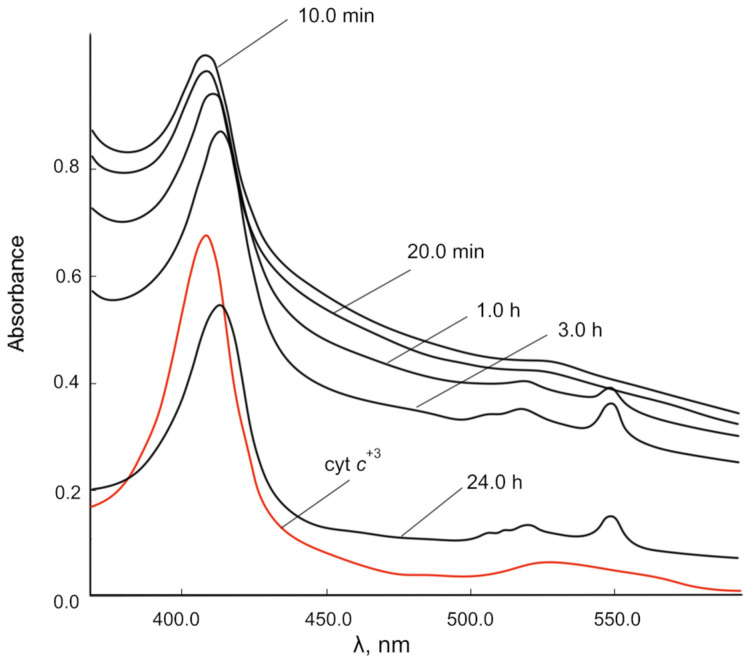
Changes in the UV-vis spectra of the reaction mixture of 0.00667 mM cyt *c*^3+^ solution over time under the action of CeO_2_ NPs (500.00 mg/L) in PBS (pH 7.4). Spectra were obtained after centrifugation of the mixtures.

Figure 7 shows the dynamics of changes in the UV-vis spectra of the reaction mixture of cyt *c*^3+^ and CeO_2_ NPs over time. The position of the maximum of the γ band shifts from 408.0 nm to 413.5 nm within 24 h, and α (549.5 nm) and β (520.5 nm) bands appear instead of a diffuse band after 1 h of exposure. Consequently, the reduced form of cyt *c* (cyt *c*^2+^), which is characterized by these spectral features (red shift of the γ band and clearly separated α and β bands), is formed under the action of nanoceria within 1 h.

A feature of the interaction of nanoceria with cyt *c* is a significant increase in the absorption of the γ band in the initial period of time, probably due to the formation of a chemisorption complex of the cyt *c* porphyrin ring with cerium ions on the nanoceria surface. The absorbance of all cyt *c*^2+^ bands decreased within 24 h, probably due to a decrease in the cyt *c* concentration and sedimentation of particles.

The interaction of cyt *c*^3+^ with the BC-CeO_2_ NPs nanocomposite containing the same concentration of CeO_2_ NPs led to similar changes in the UV-vis spectra (Figure S4). Spectra were obtained after centrifugation of the mixtures. The appearance of sharp α and β bands, as well as the red shift of the γ band, characterizing the reduced form cyt *c*^2+^ was observed after 2 h up to 24 h. The absorbance intensity of all bands did not change dramatically.

A feature of the effect of the BC-CeO_2_ NPs composite on the dynamics of the interaction with cyt *c*^3+^ is the ability of nanoceria not only to reduce cyt *c*^3+^ to cyt *c*^2+^ but also to oxidize cyt *c*^2+^ to the initial state over time (48 h). After 48 h of reaction, the γ band returned to its original position with λ = 408 nm, the α and β bands disappeared, and a diffuse band appeared in the region of 525 nm.

**Figure 8:**
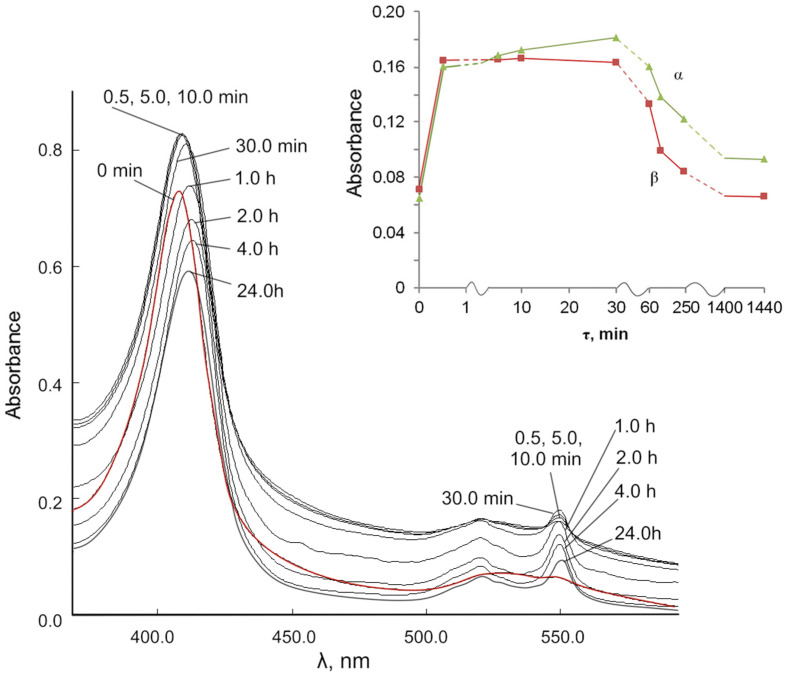
Changes in the UV-vis spectra of the reaction mixture of 0.00667 mM cyt *c*^3+^ solution over time under the action of cerium (III) nitrate (114.56 mg/L) in PBS (pH 7.4). Insert: absorbance versus time for changes in the α and β bands.

We studied the role of Ce^3+^ ions in the reduction of cyt *c*^3+^ under the action of cerium (III) nitrate in solution to exclude the effect of the dispersity of nanoceria and the BC-CeO_2_ NPs composite (Figure 8). The data on the changes in the spectra of the reaction mixture of cyt *c*^3+^ with cerium (III) nitrate in PBS (pH 7.4) characterize a clearer picture of the appearance of cyt *c*^2+^ in reduced form. The position of the γ band changes from 408.0 to 413.0 nm within 2 h and gradually decreases to 411.5 nm within 24 h (Figure 8, Table 2). There is a division of the diffuse band in the region of 520–550 nm into α and β bands during the first 0.5 min.

It is important to note that the redox nature of the interaction of cytochrome c with cerium ions in solution is similar to the redox behavior in the interaction of cyt c with the BC-CeO_2_ NPs nanocomposite (Figure S4). The inset (Figure 8) shows changes of the α and β bands in the spectra over time. The spectral data indicate the initial reduction of cyt *c*^3+^ to cyt *c*^2+^ under the action of ions Ce^3+^ and after 30–60 min, as well as the subsequent oxidation of cyt *c*^2+^ to the original cyt *c*^3+^ by Ce^4+^ ions.

We further evaluated the extent of the cyt *c*^3+^ reduction using the UV-vis spectra of homogeneous solutions of cyt *c*^3+^ and cerium (III) nitrate.

Preliminary experiments showed that in the 0.3–0.7∙10^−5^ mol/L concentration range, the dependences (A = f(C)) for solutions of cyt *c*^3+^ and cyt *c*^2+^ correspond with the Bouguer–Lambert–Beer law (Figure S5), enabling assessment of the concentration of the oxidized and reduced forms both qualitatively and quantitatively using the absorption of the γ band and the band in the region of 520–550 nm (Figure S5, Table S1).

Taking into account that in this range of concentrations and under these conditions, both the oxidized and reduced forms of cyt *c* correspond with the Bouguer–Lambert–Beer law and have a close molar extinction coefficient, it is possible, in a first approximation, to estimate the fraction of reduced cyt *c*^2+^ under the action of Ce^3+^ ions over time:

(1) $$\theta =\frac{\left|\Delta A\right|\cdot 100\%}{A_{0}},$$

where ΔA = A_0_−A_τ_, and A_0_, A_τ_ are the absorption at the initial and indicated moment of time, respectively.

An approximate evaluation of the reduced cyt *c*^2+^ fraction established by the γ band yields a value of approximately 13% in the first 10–30 min. Therefore, the experiments with cerium (III) nitrate and CeO_2_ NPs confirm the ability of CeO_2_ NPs to reduce cyt *c*^3+^ by surface Ce^3+^ ions. It is difficult to assess further changes in cyt *c*, since with a longer exposure, cerium ions can be introduced into the porphyrin cycle of cyt *c*, which causes the inhibition of the active center of cyt *c*.

We studied the participation of the obtained BC-CeO_2_ NPs in oxidation reactions using their interaction with ascorbic acid as a strong reductant in PBS (pH 7.4).

**Figure 9:**
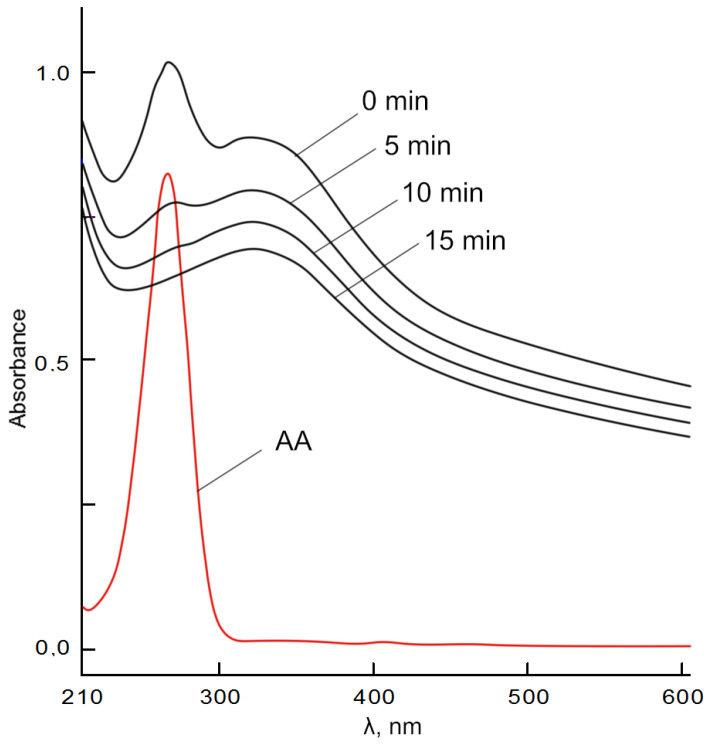
UV spectra of 0.00667 mM solution of ascorbic acid (red line) and its mixture with BC-CeO_2_ NPs in phosphate buffer solution (pH 7.4) over time. C (CeO_2_ NPs) = 0.5 mg/mL.

Figure 9 shows the UV spectrum of ascorbic acid characterized by a band with λ_max_ = 265 nm and the spectra of its mixture with BC-CeO_2_ NPs. The UV spectra of the mixture have a high-intensity band with λ_max_ = 265 nm and a shoulder in the region of 336 nm. An increase in the absorption intensity of both bands can be associated with the absorption of BC-CeO_2_ NPs in this region.

Over time, the intensity of the band at 265 nm decreased due to the π→π* transition in the enol fragment of ascorbic acid, while the absorption of the shoulder in the region of 318–336 nm increased, probably due to the n→π* and π→π* transitions of the carbonyl group. The disappearance of the absorption band in the region of 265 nm characterizes the oxidation of ascorbic acid to dehydroascorbic acid.

**Figure 10:**
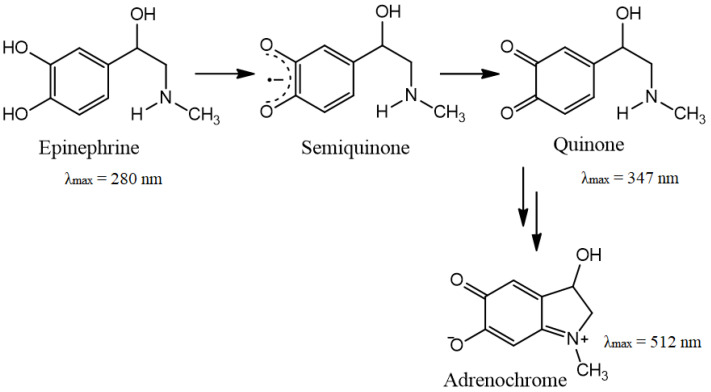
Scheme of epinephrine auto-oxidation.

The ability of CeO_2_ NPs to reduce the semiquinone anion radical, both in pure form and in the BC matrix, was studied in the auto-oxidation reactions of epinephrine hydrochloride in the presence of atmospheric oxygen in an alkaline medium. Figure 10 shows the scheme of catecholamine oxidation. The inhibition rate of the process of auto-oxidation was used as a test of SOD-like activity of nanoceria, as previously demonstrated in [34].

**Figure 11:**
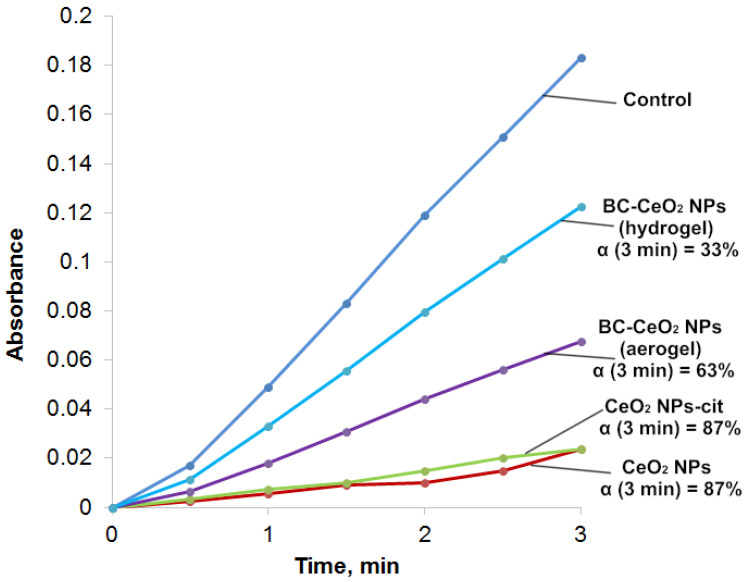
Curves of absorption (A_347 nm_) versus time for epinephrine auto-oxidation products (control) and in epinephrine dispersions with cerium oxide nanoparticles; α is calculated for 3 min.

The dynamics of absorption changes in the region of 347 nm, which characterize the formation of intermediate products of auto-oxidation of epinephrine (but not aminochrome) in the absence and under the action of CeO_2_ NPs, is shown in Figure 11. Moreover, the process of auto-oxidation of epinephrine was studied under the action of BC(aerogel)-CeO_2_ NPs.

The inhibitory effect was assessed by decreasing the absorption of reaction mixtures of epinephrine with CeO_2_ NPs or with BC-CeO_2_ NPs in the region of 347 nm compared to the control (an epinephrine solution) for 1 and 3 min. The inhibition was characterized by the relative value of α, which was calculated according to the following formula:

(2) $$\alpha =\frac{A_{0}-A_{x}}{A_{0}}\cdot 100\%,$$

where A_0_ and A_x_ are the absorption of epinephrine auto-oxidation products in the absence and presence of CeO_2_ NPs, respectively.

The absorption of the 0.256 mM solution of epinephrine hydrochloride (control) in an alkaline solution for 1–3 min was maximum, which indicates an intensive process of auto-oxidation. The absorption of epinephrine hydrochloride in the presence of CeO_2_ NPs or citrate-stabilized CeO_2_ NPs at the same time was minimal and corresponded to 13% of the uptake of the control solution. Therefore, the auto-oxidation process was inhibited by 87%. The introduction of a dispersion of BC-CeO_2_ NPs into the epinephrine solution also contributed to the inhibition of auto-oxidation, but the percentage of inhibition was lower and amounted to 63%.

### 2.3. The Study of the Effect of BC-CeO_2_ NPs on the Activity of SOD and Glucose-6-Phosphate Dehydrogenase and on Antioxidant Properties in In Vitro Experiments on Rat Blood

The activity of the antioxidant defense enzyme SOD under the action of CeO_2_ NPs and BC-CeO_2_ NPs nanoparticles was studied by evaluating the inhibition of epinephrine auto-oxidation in rat blood for 1 min [35], which is close to the described auto-oxidation experiments in PBS medium (Figure 9). SOD activity increased by about 24% compared to the control under the influence of BC-CeO_2_ NPs and CeO_2_ NPs at the same concentration (Table 3).

The antioxidant defense enzyme activity in the pentose phosphate metabolic pathway, glucose-6-phosphate dehydrogenase (G6PDH), under the action of BC-CeO_2_ NPs and CeO_2_ NPs increased more significantly (2.0–2.5 times) due to NADPH reduction by Ce^3+^ ions. The malondialdehyde level in plasma and erythrocytes either slightly decreased or remained within the control range. Other biochemical parameters characterizing the antioxidant properties of BC-CeO_2_ NPs, such as the levels of diene and triene conjugates and Schiff bases, were also close to those of the control.

## 3. Discussion

Despite the fact that many works have been devoted to the preparation of nanocomposite materials based on BC and metal oxide nanoparticles [12,18,23], the synthesis of BC–CeO_2_ NPs nanocomposites has not been widely considered. Nanocomposites are usually prepared by mixing dispersions of cellulose and other polysaccharides with stabilized CeO_2_ NPs and then forming the material [15,16,17]. This is primarily due to the method of obtaining cerium oxide nanoparticles by their precipitation from solutions of cerium (III) salts with ammonium hydroxide in a non-aqueous medium at a temperature of 40–60 °C. Bacterial cellulose in this medium is partially destroyed, with the formation of oligomeric and low-molecular-weight cellobiose fragments, which form colored complexes with cerium cations. This process proceeds especially intensively in aerogel powders with a high specific surface area (230–238 m^2^/g). In this regard, technological problems associated with the removal of degradation products and colored complexes both from BC material in the form of aerogels or films are very important.

Another technological task that arises in the formation of nanocomposites and hybrid materials based on BC and metal oxide nanoparticles is the preparation of nanoparticles of uniform size and distribution in the bulk of the material. In addition, cerium oxide nanoparticles must be stabilized immediately after production to avoid agglomeration and an increase in particle size [21,22]. Stabilization of cerium oxide nanoparticles is carried out by treatment with solutions of sodium citrate, polyethylene glycols, dextran, maltodextrin, polyacrylic acid, and other stabilizers.

The treatment of BC aerogel dispersion or BC film with cerium nitrate in an aqueous-ethylene glycol medium ensures uniform sorption of Ce^3+^ ions in the polymer matrix for a fairly short time at room temperature. The formation of cerium oxide nanoparticles under the action of trisamine in the BC matrix makes it possible to form 2–5 nm nanoparticles stabilized by cellulose units. The advantage of the proposed method is the simplicity of removal of the initial reagents and degradation products of cellulose.

In addition, the trisamine (trioxymethylaminomethane) precursor is an API used as a systemic antacid. Trisamine exhibits buffering properties, eliminates intracellular acidosis, exhibits osmotic and diuretic effects, and is excreted unchanged by the kidneys. Trisamine is used in procedures accompanied by metabolic and mixed acidosis, which occur during burns and in the healing of complex wounds.

The “soft” method proposed in this work to obtain BC-CeO_2_ NPs nanocomposite materials using cerium nitrate and organic amine (trisamine) as precursors in an aqueous-ethylene glycol medium at room temperature enables the attainment of nanocomposites with a controlled concentration of cerium oxide nanoparticles amounting to 0.2–60.0%. Although Ce^3+^ ions are non-covalently bound with monomeric units of cellulose (structures Ia, Ib, and Ic in Figure 1 and structures IIa, IIb, and IIc in Figure 2), trisamine enables the oxidation of Ce^3+^ to cerium (IV) hydroxide and hydrated CeO_2_ NPs nanoparticles in the BC matrix without the formation of colored complexes of destructed BC units.

This technique makes it possible to obtain new materials based on BC both in the form of a hydrogel film and in the form of aerogel powder. Identification of cerium oxide nanoparticles in the BC matrix using the PXRD method showed a cubic structure of the fluorite of cerium oxide nanoparticles in the material that was close to the structure of individual CeO_2_ NPs obtained by the deposition method. FTIR, SEM, and EDX methods revealed a uniform distribution of nanoparticles in the composite. The particle size of CeO_2_ NPs in the resulting BC-CeO_2_ NPs composites did not change over the course of a year, in contrast to pure and sodium-citrate-stabilized CeO_2_ NPs, as estimated by PRXD and SEM data. Samples should be stored in a dark, dry place at 15–30 °C to avoid water absorption, since BC sorbs moisture intensively.

To study the redox properties of CeO_2_ NPs in the polymer matrix, we used known methods for assessing the SOD-mimetic activity [36]. The most important tests were studies of reactions of CeO_2_ NPs with cytochrome *c* as an indicator of SOD-mimetic activity, as well as tests of the inhibition of epinephrine auto-oxidation reactions described in [34]; we also used a standard reaction with ascorbic acid as a test.

As follows from the results presented in Figure 11 and Table 3, the CeO_2_ NPs obtained by us in a composite material exhibit SOD-mimetic activity and redox properties close to those pure CeO_2_ NPs and those reported in the literature data [37].

The high Ce^3+^ concentration in CeO_2_ NPs (30–40%) in the BC-CeO_2_ NPs composite suggests the participation of Ce^3+^ ions and CeO_2_ NPs in reduction reactions. The presence of Ce^4+^ ions in CeO_2_ NPs at a high concentration (60–70%) promotes oxidation reactions. The autoregeneration of CeO_2_ NPs can be assumed, which is similar in nature to oscillatory reactions, under the action of cytochrome *c*, in contrast to traditional antioxidants, such as vitamins C and E.

Cytochrome *c* (cyt *c*), as a heme-containing ferroprotein, participates in electron transport in the breathing chain. Moreover, an oxidized form of cyt *c* (Fe^3+^) activates caspase and leads to programmed cell death, while the reduced form (Fe^2+^) cannot do so. Cytosolic cyt *c* of healthy cells is rapidly reduced by various enzymes or reductant species, which blocks apoptosis, whereas in apoptotic cells, the oxidized cytosolic cyt *c* is rapidly oxidized by mitochondrial cytochrome oxidase and accessible due to the permeability of the outer membrane [29,30]. The effect of Ce^3+^ and Ce^4+^ cerium ions on the redox state of cyt *c* is significant, given its regulatory function in the human body, and cyt *c* is a convenient marker of redox reactions under the action of CeO_2_ NPs.

It can be assumed that the inhibition of epinephrine auto-oxidation under the action of CeO_2_ NPs and BC-CeO_2_ NPs is probably due to the reduction of the catecholate radical anion to the original epinephrine, similarly to the reduction of the superoxide anion by superoxide dismutase (SOD).

In general, it can be noted that BC-CeO_2_ NPs composites exhibit antioxidant properties, regulate the redox state of CeO_2_ NPs in the BC matrix, and have SOD-mimetic properties that enable the design of new antioxidant drugs to treat various diseases.

## 4. Materials and Methods

### 4.1. Preparation of BC

BC was produced in a static culture medium by *Komagataeibacter sucrofermentans* H-110, which was isolated from Kombucha tea and identified by sequencing of the amplified product of 16S rRNA [38,39].

### 4.2. Synthesis of Cerium Oxide Nanoparticles

#### 4.2.1. Synthesis of Cerium Oxide Nanoparticles in Ethylene Glycol

Synthesis was carried out according to the procedure described in [27] with minor modifications. Briefly, cerium nitrate hexahydrate (2.175 g, 5 mmol) was heated to 60 °C in 100 mL of ethylene glycol–water solution (7:3) in a flask; then, 27% aqueous ammonium hydroxide was added dropwise very slowly to pH 10–11. The resulting yellow–beige precipitate was centrifuged, washed with hot water and ethanol several times, and dried under vacuum.

#### 4.2.2. Synthesis of Cerium Oxide Nanoparticles in a BC Matrix

At the first stage of CeO_2_ NPs synthesis in a BC matrix (aerogel powder), the sorption of cerium (III) nitrate (1–50 mmol) was carried out using BC (0.2–0.8 g) in a solvent mixture (ethylene glycol–water = 7:3). After washing with ethanol, BC was transferred to solution (ethylene glycol–water = 7:3). Then, hydrolysis of cerium (III) nitrate sorbed on BC with 27% ammonium hydroxide solution was performed at 60 °C. BC samples were thoroughly washed and dried. The disadvantage of BC-CeO_2_ NPs synthesis using this method is the difficulty in removing various colored (from blue–violet to brown tones) complexes of cerium with a monomeric unit of cellulose and ammonium hydroxide.

Alternative method: The BC aerogel powder (or BC hydrogel film) was kept in ethylene glycol–water (7:3) for 10–24 h. BC was immersed in a solution of cerium (III) nitrate for 1–3 h; then, the material was carefully washed with water and ethanol. The wet material was immersed in a container with a 0.1–1.0 M trisamine solution for 1 h, then carefully washed with water and ethanol. The resulting material was dried under vacuum.

### 4.3. FTIR Analysis

FTIR spectra in the range of 400–4000 cm^−1^ were registered by an IR Prestige-21 FTIR spectrometer (Shimadzu, Kyoto, Japan) equipped with a KBr beam splitter. A pellet from a well-dried KBr was prepared by standard cold pressing. The resolution was 0.5 cm^−1^, and the number of scans was 45.

### 4.4. UV Analysis

UV spectra were obtained by UV-1800 (Shimadzu, Kyoto, Japan). PBS was used as a solvent. The wavelength range depended on the analyzed substance.

### 4.5. Powder X-Ray Diffraction Analysis

Powder X-ray diffraction patterns were registered using a Shimadzu XRD-6000 X-ray diffractometer (Shimadzu, Kyoto, Japan) at 295(2) K with Cu Kα radiation (λ = 1.5418 Å) in the Bragg–Brentano reflection geometry. The samples were collected in the 2θ range between of 5 to 50° with steps of 0.026° and a 100 s step size, using a scan speed (°/s) of 0.067335. On the X-ray diffraction patterns of amorphous samples, there were diffraction peaks at 37.5° and 44.0° referring to the cuvette material.

### 4.6. SEM Analysis

The morphology of the samples was obtained by scanning electron microscopy (SEM) on a JSM-IT300LV (JEOL, Tokyo, Japan) with a electron probe diameter of about 5 nm and a probe current below 0.5 nA (operating voltage: 20 kV). The sample surface topography was studied using low-energy secondary electrons and backscattered electrons under low vacuum mode to eliminate charging.

### 4.7. Specific Surface Area Analysis

Specific surface areas (SSAs) of the CeO_2_ NPs were analyzed using an ASAP 2020 analyzer of specific surface area and adsorption porosity (Micromeritics, Norcross, GA 30093, USA).

### 4.8. Chemical Composition of BC

The chemical composition of BC was analyzed by determining C/O/N using a Flash EA 1112 CN analyzer (NEOLAB Ltd., Florence, Italy). Analysis of major and trace elements was performed with an AA-7000 atomic absorption spectrophotometer (Shimadzu, Kyoto, Japan) after wet mineralization of cellulose samples with a mixture of perchloric and nitric acids.

### 4.9. Evaluation of Epinephrine Auto-Oxidation Inhibition

A volume of 0.2 mL of 0.1% (5.46 mM) epinephrine hydrochloride solution was added to 4 mL of 0.2 M sodium carbonate buffer (pH 10.65) (set by adding dry NaHCO_3_ reagent to 0.2 M Na_2_CO_3_ solution), then carefully and quickly mixed. The solution absorption was determined every 30 s for 10 min at a wavelength of 347 nm in a 10 mm thick (A_0_) cuvette. Then, 0.2 mL of 0.1% epinephrine hydrochloride and 0.06 mL of the test sample were added to 4 mL of buffer (pH 10.65) and mixed, and optical density was measured as described above (A_x_).

### 4.10. Biological Activity

This study involved male Wistar rats (150–200 g). The animals were purchased from the Animal Breeding Facilities of “Andreevka”, Federal State Budgetary Institution of Science “Scientific Center for Biomedical Technologies”, Federal Medical and Biological Agency (Andreevka, Moscow region, Russia). All procedures for maintenance and sacrifice (care and use) of animals were carried out according to the criteria outlined by European Convention ET/S 129, 1986 and directives 86/609 ESC. The animals were handled humanely, kept in plastic suspended cages, and placed in a well-ventilated and hygienic rat house under suitable conditions of room temperature (27 ± 2 °C) and humidity. They were given food and water ad libitum and subjected to a natural photoperiod cycle of 12 h light and 12 h dark. The animals were allowed two weeks of acclimatization before the commencement of all animal model experiments in the study.

All blood collection from animals for the experiment was performed under anesthesia, with efforts made to minimize suffering.

The study, as presented, was approved by the Local Ethics Committee of Privolzhsky Research Medical University, Russian Federation (Protocol No. 1, 18 January).

In vitro biological analysis was performed using blood stabilized with sodium citrate (1:9). Erythrocytes were washed twice with 0.9% NaCl by centrifugation for 10 min at 1600× *g*. The intensity of lipid peroxidation (LPO) was estimated using the MDA level in plasma and erythrocytes in accordance with the methods of Uchiyama and Mihara [40]. The contents of diene conjugates (DC), triene conjugates (TC), and Schiff bases (SB) in the blood plasma were determined out according to the Khyshiktuev method [41]. Superoxide dismutase (SOD) activity (EC 1.15.1.1) was measured in erythrocytes using the inhibition of epinephrine auto-oxidation [36]. The activity of glucose-6-phosphate dehydrogenase (G6PDH) (EC 1.1.1.49) was determined in accordance with the method described in [42].

### 4.11. Statistical Analysis

Statistical data processing was performed using Statistica 6.0 software (StatSoft Inc., Tulsa, OK, USA). The normality of the distribution of the results was shown using the Shapiro–Wilk test. The significance of differences between groups was assessed using Student’s t-test and one-way analysis of variance (ANOVA). Differences were considered statistically significant at *p* < 0.05.

If the distribution of at least one of the populations was not normal, non-parametric analysis methods were used for comparison. The nonparametric Kruskal–Wallis test was used for multiple comparisons of independent groups. The Mann–Whitney test was used for multiple comparisons for 2 groups.

## 5. Conclusions

An important constituent in creating BC-based nanocomposites is to confirm the redox properties of cerium oxide nanoparticles in the polymer matrix, i.e., BC. The inhibition of auto-oxidation of epinephrine, the oxidation of ascorbic acid, and redox reactions of cytochrome c are convenient express tests for evaluating the redox properties of CeO_2_ NPs in the BC matrix and the BC-CeO_2_ NPs composite.

It has been shown that CeO_2_ NPs in the BC matrix can be considered to exhibit the properties of an SOD mimetic, which is important when preparing drugs to treat the diseases caused by ROS generation and oxidative stress. In the present study, SOD-mimetic properties were confirmed by a dramatic improvement in the activity of two enzymes of antioxidant defense, i.e., SOD and G6PDH.

## Figures and Tables

**Table 1:** Data of powder XRD patterns of BC-CeO_2_ NPs samples (Figure 3).

Figure Number	Composition	2θ, Degree	D, nm	Structure Signal
a	CeO_2_ NPs	28.32	3.26	CeO_2_
		32.58	3.50	
		46.82	3.02	
		56.04	2.90	
b	BC ^1^	22.96	4.75	BC
c	BC(aerogel)-CeO_2_ NPs	21.88	3.50	BC
		28.42	3.50	CeO_2_
		32.24	5.00	
		47.08	3.00	
		55.58	3.43	
d	BC(hydrogel)-CeO_2_ NPs	22.18	3.80	BC
		28.32	3.60	CeO_2_
		33.58	3.00	
		46.94	3.00	
		56.24	3.00	

^1^ Data of our samples of BC were published in [28].

**Table 2:** Data of UV-vis spectra of 0.00667 mM cyt *c*^3+^ solution with cerium (III) nitrate (114.56 mg/L) in PBS (pH 7.4).

τ, min	Band								
	γ					β		α	
	A	ΔA	θ, %	λ, nm	|Δλ|, nm	A	λ, nm	A	λ, nm
0	0.729	-	0	408.0	-	-	-	-	-
0.5	0.828	0.099	13.6	408.5	0.5	0.165	520.5	0.161	549.0
5.0	0.829	0.100	13.7	409.0	1.0	0.165	520.5	0.168	549.5
10.0	0.826	0.097	13.3	409.0	1.0	0.166	520.5	0.172	549.5
30.0	0.811	0.082	11.2	410.5	2.5	0.163	520.0	0.181	549.5
60.0	0.739	0.010	1.4	412.0	4.0	0.133	520.0	0.160	549.0
120.0	0.681	−0.048	N/a *	412.5	4.5	0.099	520.0	0.138	549.5
240.0	0.645	−0.084	N/a	413.0	5.0	0.084	520.0	0.122	549.5
1440.0	0.593	−0.136	N/a	411.5	3.5	0.066	520.5	0.093	549.5

* N/a—not available; the cyt *c*^3+^ structure is changed under these conditions.

**Table 3:** Biochemical indices in rat blood under the influence of CeO_2_ and BC-CeO_2_ NPs at a concentration of 50 μL of the sample per 1 mL of blood (in % of the control; *n* = 3).

Sample	Biochemical Index *						
	SOD, inh/min*mg of Protein	G6FDH, NADPH/min*mg of Protein	MDA_pl_, μmol/L	MDA_er_, μmol/L	DC, Arbitrary Units	TC, Arbitrary Units	SB, Arbitrary Units
Control (100%)	962.14 ± 12.54	38.12 ± 3.54	1.03 ± 0.04	7.26 ± 0.84	0.76 ± 0.03	0.26 ± 0.01	0.14 ± 0.01
CeO_2_NPs	1229.24 ± 43.98	104.53 ± 5.73	0.93 ± 0.02	6.53 ± 0.22	0.68 ± 0.02	0.25 ± 0.01	0.14 ± 0.01
BC-CeO_2_ NPs	1198.63 ± 23.09	67.88 ± 1.24	0.86 ± 0.01	7.42 ± 0.67	0.65 ± 0.02	0.25 ± 0.01	0.13 ± 0.01

* Kruskal–Wallis test: *p* ≤ 0.0001.

## Data Availability

The data presented in this study are available upon reasonable request from the corresponding author.

## Supplementary Materials

The following supporting information can be downloaded at: https://www.mdpi.com/article/10.3390/molecules28062604/s1, Figure S1: FTIR spectrum of CeO_2_ NPs synthesized in ethylene glycol medium; Figure S2: PXRD pattern of CeO_2_ NPs, ICDD-JCPDS database (JCPDS No. 34-0394); Figure S3: SEM and EDX data of CeO_2_ NPs; Figure S4: UV spectra of 0.0666 mM solution of cyt *c*^3+^ with BC-CeO_2_ NPs (7 mg in 5 mL of phosphate buffer solution). Spectra were obtained after centrifugation of mixtures. Data of the UV spectrum of Blank cyt *c*^2+^ solution—λ, nm/A = 414.0 nm/0.708; 520.0 nm/0.082; 549.5 nm/0.113; Figure S5: UV spectra of cyt *c*^3+^ (a) and cyt *c*^2+^ (b) in phosphate buffer solution, pH 7.4; Table S1: Data of UV-vis spectra of cyt *c*^3+^ and cyt *c*^2+^ in phosphate buffer solution, pH 7.4.

## Sample Availability

Samples of BC are available from the authors.
