# Supplementary material for: Synthesis of Cerium Oxide Nanoparticles in a Bacterial Nanocellulose Matrix and the Study of Their Oxidizing and Reducing Properties
Source: Molecules. 2023 Mar 13;28(6):2604. doi: 10.3390/molecules28062604 (PMC10051255; doi:10.3390/molecules28062604)
Supplement: Supplementary file 1 [file molecules-28-02604-s001.zip › molecules-2222117-supplementary.pdf]

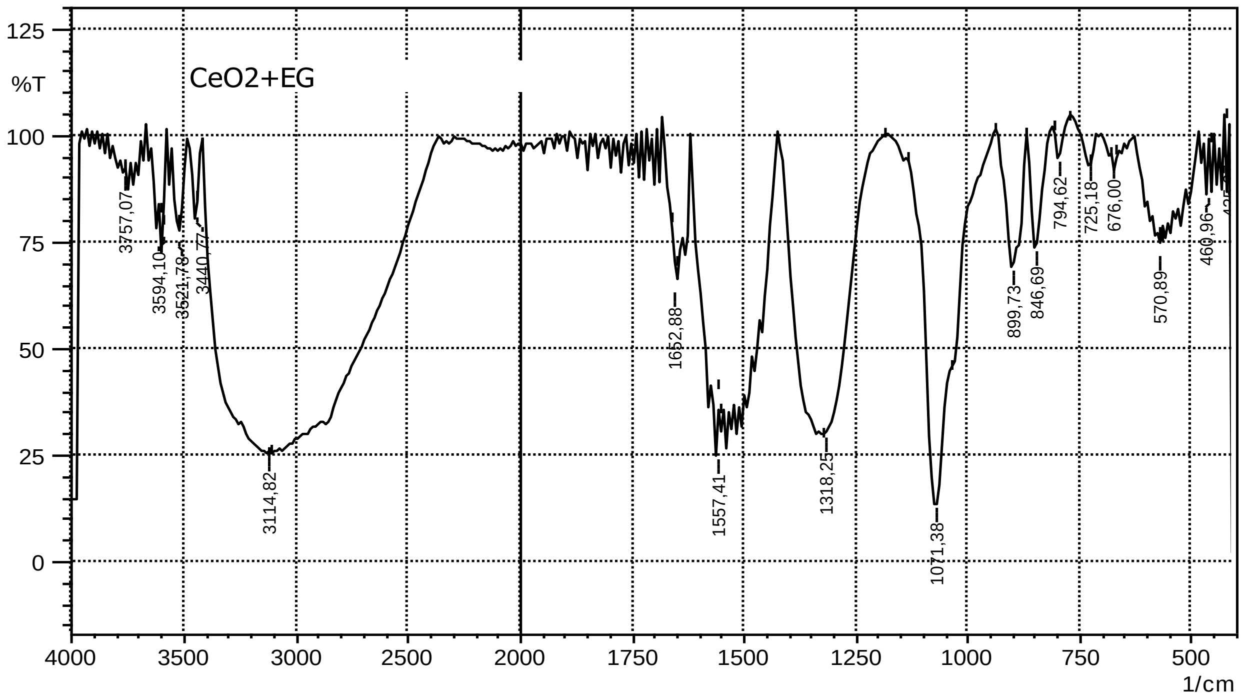

Peak	T,% (functional group)
425.27	97.847 (Ce)
460.96	86.456 (Ce)
570.89	72.894 (Ce)
676.00	94.199
725.18	93.856
794.62	94.813
846.69	73.958
899.73	69.217
1071.38	13.384 (alcohol O-H)
1318.25	29.987
1557.41	24.743 (impurity of NO $^{3-}$)
1652.88	63.899
3114.82	25.599
3440.77	81.946 (OH)
3521.78	76.108 (OH)
3594.10	77.472 (OH)
3757.07	91.649 (OH)

Figure S1. FTIR spectrum of CeO_2 NPs synthesized in ethylene glycol medium.

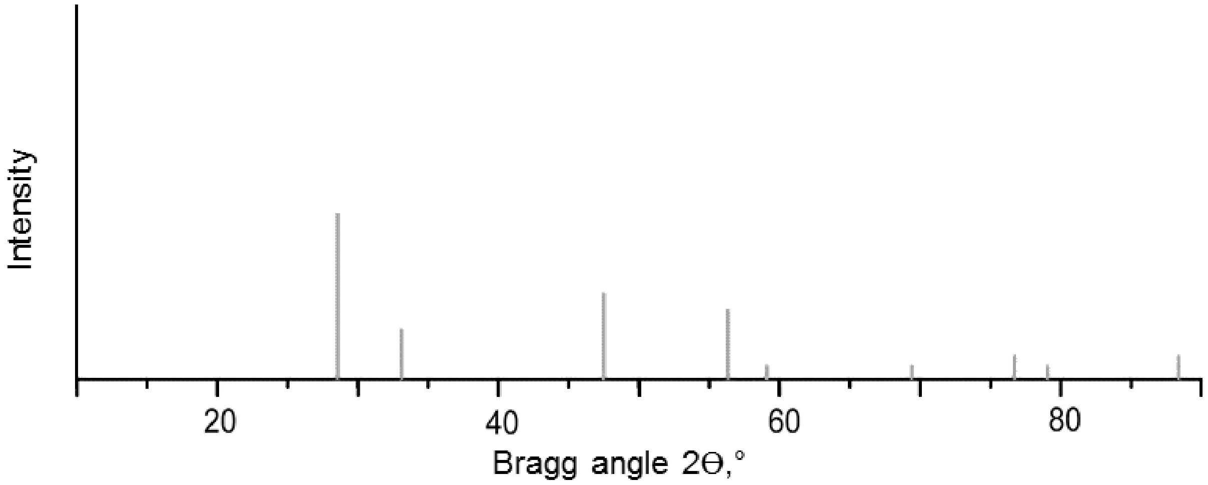

Figure S2. PXRD pattern of CeO_2 NPs, ICDD-JCPDS database (JCPDS No. 34-0394).

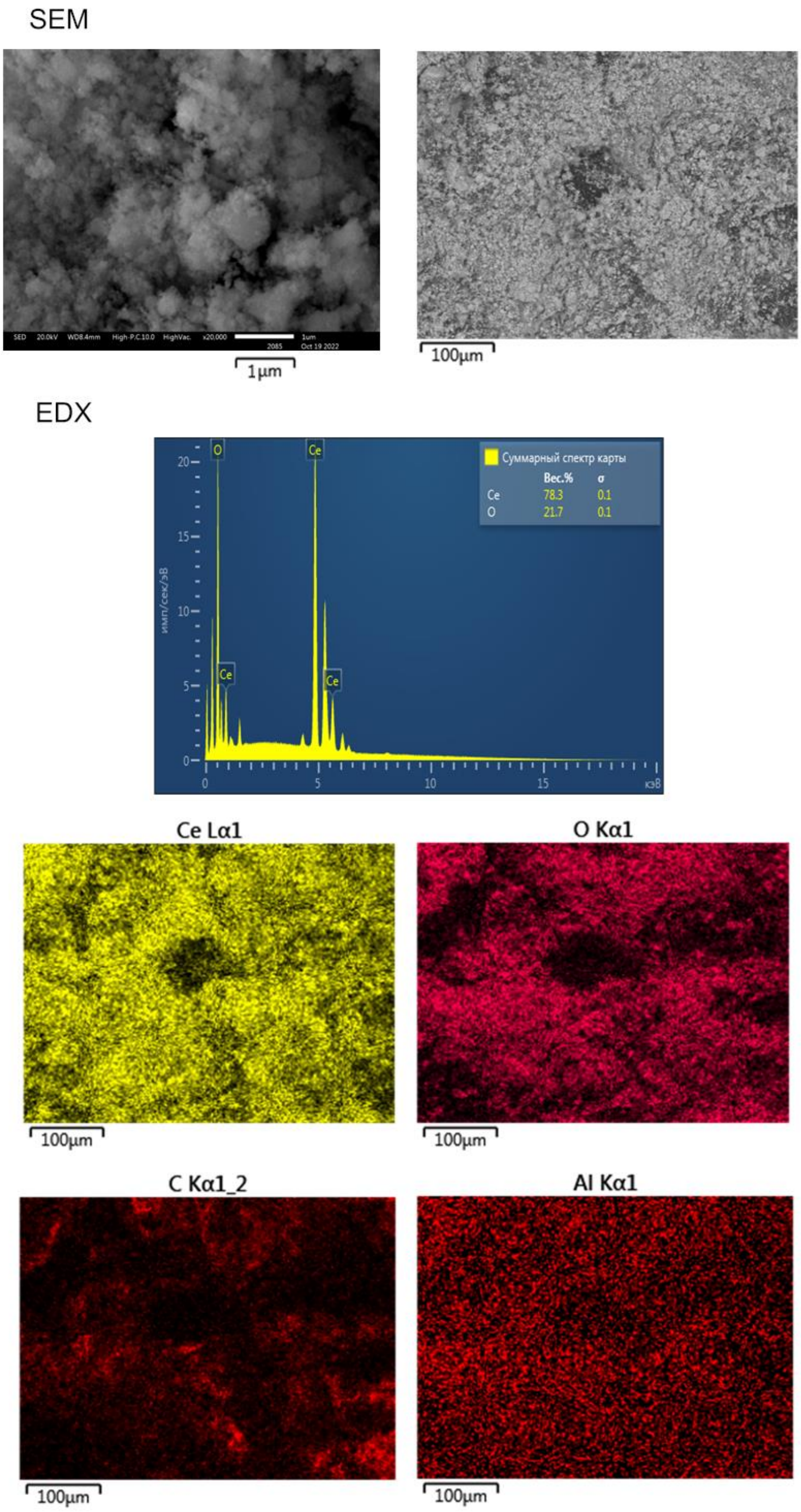

Figure S3. SEM and EDX data of CeO_2 NPs.

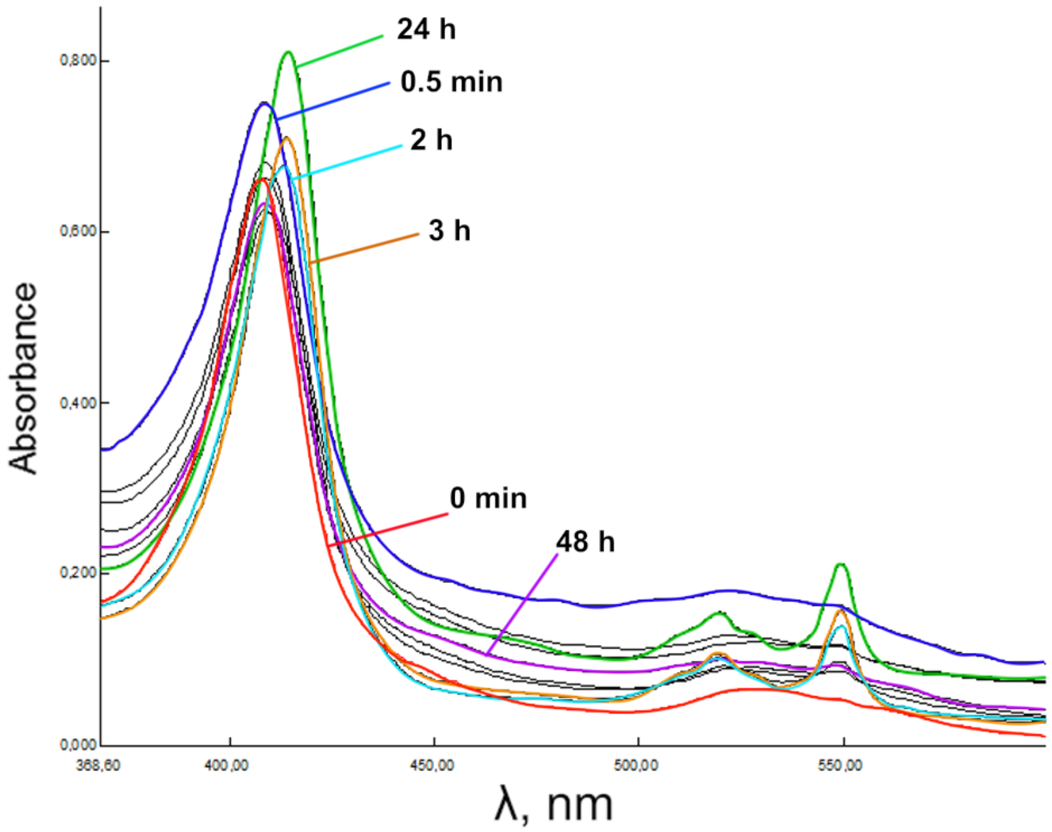

No		τ, min	Band	
			γ	
			A	λ, nm
1		0	0.663	408.0
2		0.5	0.752	408.5
3		5.0	0.680	409.0
4		10.0	0.663	409.0
5		30.0	0.634	409.0
6		60.0	0.625	409.0
7		120.0	0.683	413.5
8		180.0	0.710	414.0
9		1440.0	0.810	414.5
10		2880.0	0.626	408.0

Figure S4. UV spectra of 0.0666 mM solution of cyt c^{3+} with BC-CeO_2 NPs (7 mg in 5 ml of phosphate buffer solution). Spectra were obtained after centrifugation of mixtures.
Data of the UV spectrum of Blank cyt c^{2+} solution – λ, nm/A = 414.0 nm/0.708; 520.0 nm/0.082; 549.5 nm/0.113.

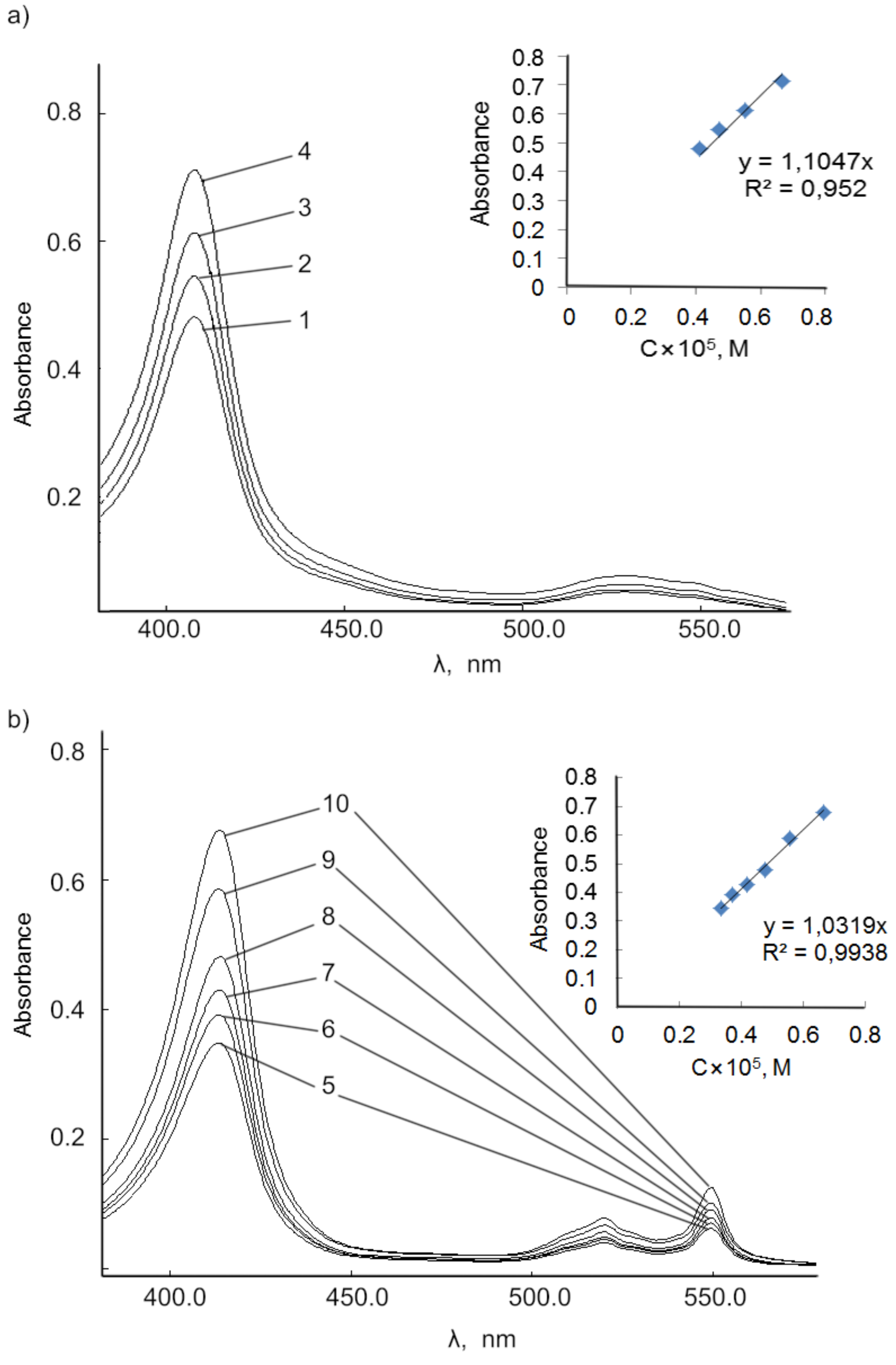

Figure S5. UV spectra of cyt c^{3+} (a) and cyt c^{2+} (b) in phosphate buffer solution, pH 7.4.

Table S1. Data of UV-vis spectra of cyt c^{3+} and cyt c^{2+} in phosphate buffer solution, pH 7.4.

Sample	Curve	$C \times 10^5$, M	λ, nm	A (γ)	ε $L \cdot mol^{-1} \cdot cm^{-1}$
**Cyt c^{+3} (Fig. S4a)**	**1**	0.416	408.0	0.480	115384.6
	**2**	0.475	408.0	0.544	114526.3
	**3**	0.555	408.0	0.611	110090.1
	**4**	0.666	408.0	0.711	106756.8
**Cyt c^{+2} (Fig. S4b)**	**5**	0.333	413.5	0.347	104204.2
	**6**	0.370	413.5	0.391	105675.7
	**7**	0.416	414.0	0.429	103125.0
	**8**	0.475	414.0	0.481	101263.2
	**9**	0.555	413.5	0.586	105585.6
	**10**	0.666	414.0	0.676	101501.5
